# RETRACTION: Circular RNA SMARCA5 May Serve as a Tumor Suppressor in Non‐Small Cell Lung Cancer

**DOI:** 10.1002/jcla.70243

**Published:** 2026-04-13

## Abstract

**RETRACTION**: S. Tong, “Circular RNA SMARCA5 May Serve as a Tumor Suppressor in Non‐Small Cell Lung Cancer,” *Journal of Clinical and Laboratory Analysis* 34, no. 5 (2020): e23195, https://doi.org/10.1002/jcla.23195.

The above article, published online on 16 January 2020 in Wiley Online Library (wileyonlinelibrary.com), has been retracted by agreement between the journal Editor‐in‐Chief, Rong Fu; and Wiley Periodicals, LLC. The retraction has been agreed due to concerns raised by a third party, which identified compromised data and systematic issues in the published article. Particularly, during the investigation, relevant inconsistencies regarding the patient data was identified. The authors and their institutes were unresponsive when these concerns were brought to their attention. Accordingly, the article is retracted as the editors have lost trust in the reliability of the data and the results presented.

**RETRACTION**: S. Tong, “Circular RNA SMARCA5 May Serve as a Tumor Suppressor in Non‐Small Cell Lung Cancer,” *Journal of Clinical and Laboratory Analysis* 34, no. 5 (2020): e23195, https://doi.org/10.1002/jcla.23195.

The above article, published online on 16 January 2020 in Wiley Online Library (wileyonlinelibrary.com), has been retracted by agreement between the journal Editor‐in‐Chief, Rong Fu; and Wiley Periodicals, LLC. The retraction has been agreed due to concerns raised by a third party, which identified compromised data and systematic issues in the published article. Particularly, during the investigation, relevant inconsistencies regarding the patient data was identified. The authors and their institutes were unresponsive when these concerns were brought to their attention. Accordingly, the article is retracted as the editors have lost trust in the reliability of the data and the results presented.

